# A case report of a MODY6 patient coexistence with Charcot-Marie-Toothe 1A syndrome

**DOI:** 10.3389/fendo.2025.1502783

**Published:** 2025-02-14

**Authors:** Jianyu Wang, Chunhua Wang, Yujie Chen, Shuang Qi, Min Wang

**Affiliations:** ^1^ Department of Health Management Center, Qilu Hospital of Shandong University Dezhou Hospital (Dezhou People’s Hospital), Shandong, China; ^2^ Department of General Practice, Qilu Hospital of Shandong University Dezhou Hospital (Dezhou People’s Hospital), Shandong, China

**Keywords:** diabetes, *NEUROD1* mutation, MODY6, *PMP22*, CMT1A

## Abstract

Monogenic diabetes, which encompasses neonatal diabetes (NDM), maturity onset diabetes of the young (MODY), and several diabetes-associated syndromes, primarily arises from impaired function or abnormal development of the islets of Langerhans, particularly pancreatic β-cells responsible for insulin secretion. This condition is typically associated with a single pathogenic genetic mutation. Charcot-Marie-Tooth disease type 1A (CMT1A) is a hereditary demyelinating neuropathy that is caused by a duplication of the PMP22 gene located on chromosome 17. Herein, we report a case of a young Chinese patient with MODY6 harboring a novel mutation (c. 317C>T, p. Ala106Val) in the *NEUROD1* gene. Additionally, this patient concurrently presents with CMT1A, which is characterized by a large segmental duplication within the exon of the *PMP22* gene and its adjacent regions. Considering the patient’s compromised islet function, we treat him with insulin and oral hypoglycemic agents (metformin, acarbose). This represents the first reported instance of a patient with NEUROD1-MODY coexisting with CMT1A.

## 1 Introduction

MODY6, a rare subtype of MODY, results from either a heterozygous or homozygous mutation in the NEUROD1 gene on chromosome 2q32 (1, 2). NEUROD1 functions as a transcription factor that binds to and activates the insulin promoter, playing a crucial role in maintaining normal glucose homeostasis (3). MODY should be strongly suspected in individuals under 35 years of age (with those under 25 being particularly indicative) who present with mild hyperglycemia at onset and lack the typical features of either type 1 or type 2 diabetes mellitus (2, 4). The clinical manifestations of MODY6 encompass a broad spectrum, varying from patients displaying typical MODY characteristics to those who resemble individuals with common type 2 diabetes mellitus (5). The two primary sequencing technologies used for genetic diagnosis of MODY are targeted sequencing of genes associated with monogenic diabetes and whole exome sequencing (6).

Hereditary motor and sensory neuropathies, commonly referred to as Charcot–Marie–Tooth disease (CMT), are marked by a length-dependent loss of axonal integrity in the peripheral nervous system (PNS), leading to progressive muscle weakness and sensory deficits. The genetic diagnosis of CMT has been facilitated by targeted next-generation sequencing and whole-exome sequencing methods. CMT1A, the most common form of Charcot-Marie-Tooth disease (CMT), is caused by an approximately 1.4 Mb duplication of the PMP22 gene on chromosome 17p11.2-12. This genetic alteration leads to disrupted myelin formation and impaired nerve function. Most patients with CMT1A exhibit a ‘typical’ phenotype, which includes onset in childhood, sensory loss, distal weakness, absent reflexes and foot deformities (7, 8).

Herein, we presented a MODY6 patient coexistence with Charcot-Marie-Toothe 1A syndrome. The whole-exome sequencing (WES) revealed a novel mutation in NEUROD1 (c. 317C>T, p. Ala106Val), along with an approximately 1.38Mb duplication (copy number: 3) in the patient’s 17p12 region, which encompasses the PMP22 gene. As the first reported case of a patient with NEUROD1-MODY coexisting with CMT1A, it exerts positive effects on improving clinicians’ understanding of MODY6 coexisting with CMT1A.

## 2 Case description

**Figure 1:**
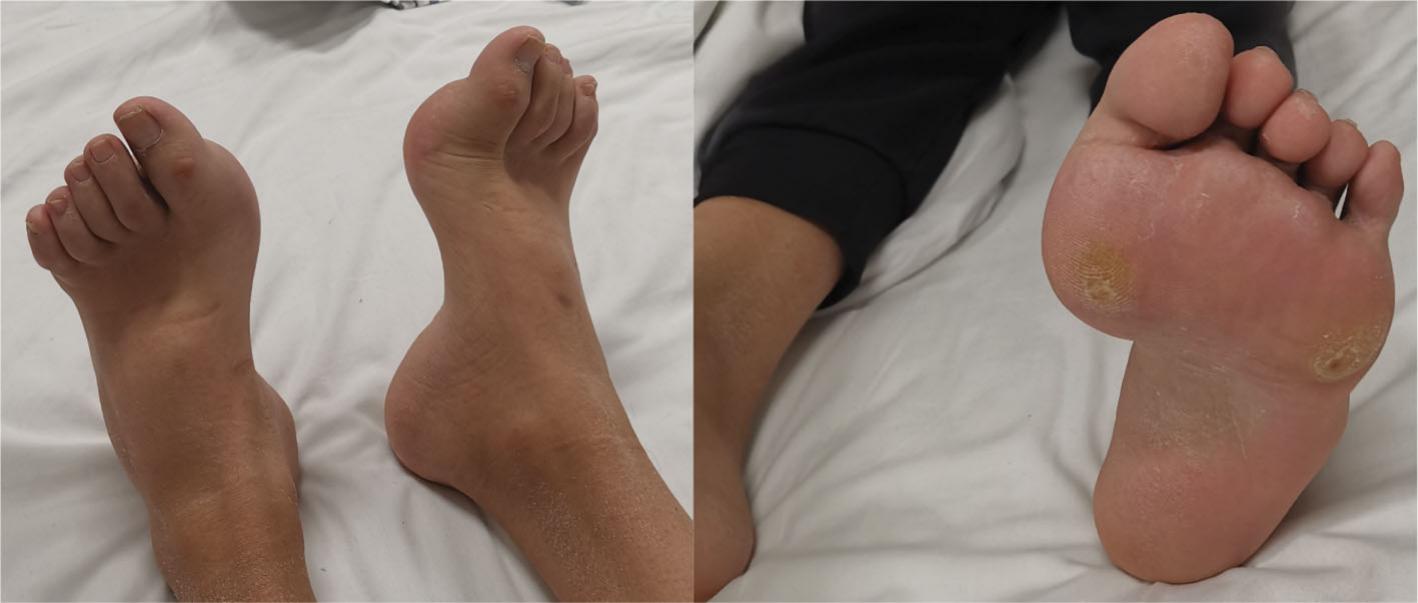
The images of patient’s high arch.

**Figure 2:**
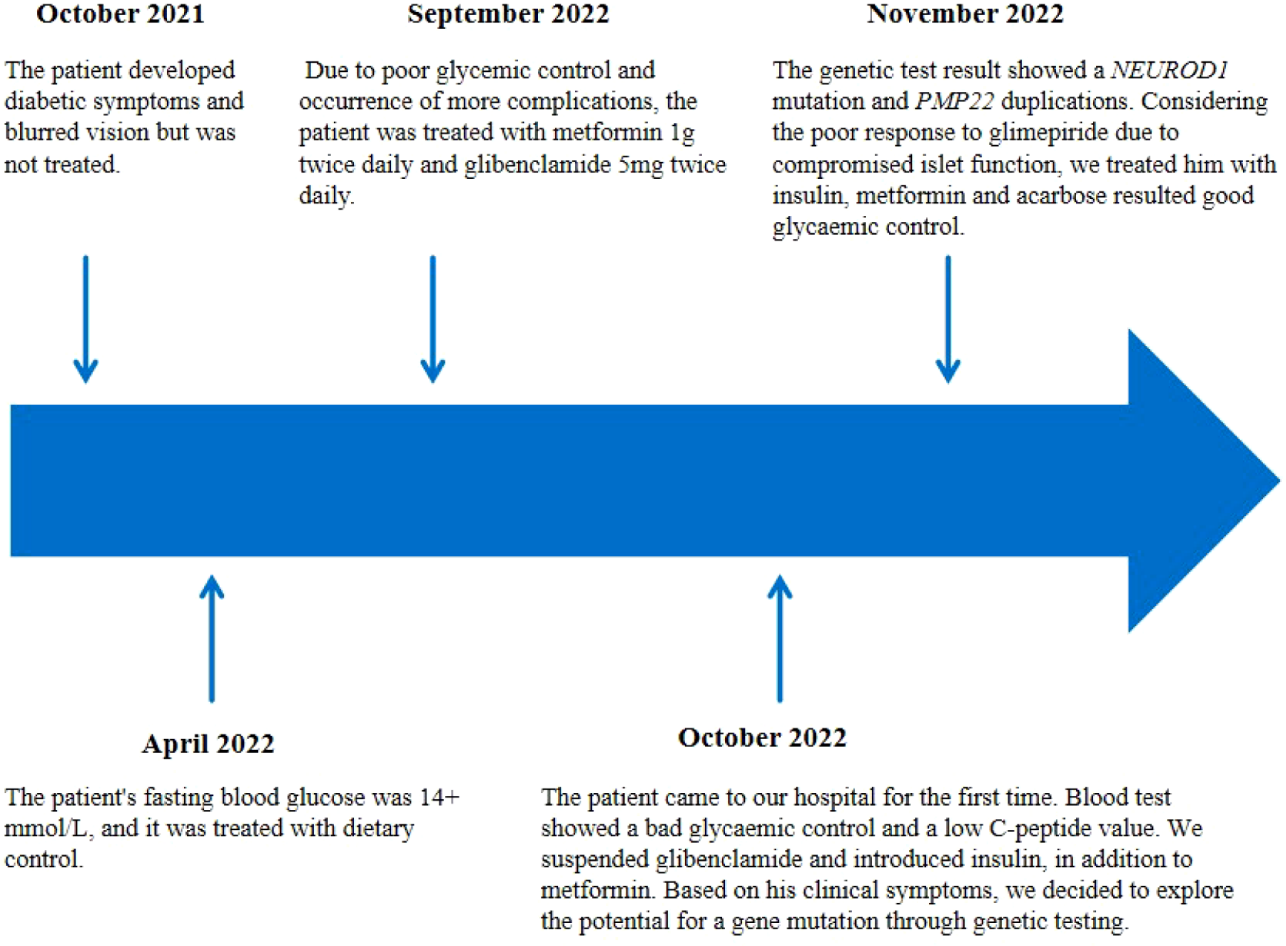
The timeline of the patient’s care.

A 29-year-old male was admitted to QiLu hospital of Shangdong university Dezhou hospital (Dezhou People’s Hospital), presenting with a one-year history of polydipsia, polyuria, and blurred vision. Six months prior, the patient’s fasting blood glucose level was measured at 14+ mmol/L at a local community outpatient clinic. However, he did not accept any treatment and did not monitor blood glucose. Ten days ago, the patient reported experiencing numbness in the hands and feet and had a fasting blood glucose level measured at over 16 mmol/L at a local community outpatient clinic. Treatment with “metformin 1g twice daily and glibenclamide 5mg twice daily” was initiated to manage blood glucose levels, yet hyperglycemia persisted. Anamnestic history revealed progressive atrophy and weakness of the distal extremities, resulting in unsteady gait since childhood. Physical examination revealed a high arch, as shown in **Figure 1**, accompanied by numbness in the limbs and decreased sensation to pinprick and temperature in the lower extremities. Electromyography: The motor nerve of left and right common peroneal nerves and tibial nerve are not elicited, the sensory nerve of left and right superficial peroneal nerves are injured, the sensory nerve amplitude of left and right sural nerves reduced. The family history: The patient’s mother was diagnosed with diabetes at the age of 50, with a BMI of 24kg/m^2^. She was treated with metformin 500mg once daily and maintained normal blood glucose levels. Additionally, the patient’s father also exhibited high arch and decreased running ability, while his son developed a high arch at the age of 2. The clinical information was collected and recorded by the QiLu hospital of Shangdong university Dezhou hospital (Dezhou People’s Hospital). The patient is described in **Table 1** and the timeline of the patient’s care is showed in **Figure 2**.

**Table 1:** Clinical characterization about diabetes of the patient.

	At diagnosis
Age (years)	29
BMI (kg/m2)	21
FBG (mmol/L)	9
C-Peptide (ng/ml)	0.55
HbA1c (%, mmol/mol)	12.7
Symptoms of diabetes	polydipsia, polyphagia, polyuria
acanthosis nigricans	(-)
IAA	(-)
ICA	(-)
GABA	(-)
Urine ketones	(-)
UCAR (mmol/L)	24.26
TC (mmol/L)	4.48
TG (mmol/L)	1.17
LDL (mmol/L)	3.19
VLDL (mmol/L)	1.19

BMI, body mass index; FBG, fasting blood glucose; IAA, Insulin autoantibodies; ICA, islet cell antibody; GADA, glutamate decarboxylase antibody; UCAR, urine albumin creatine ratio; TC, cholesterol; TG, triglycerides; LDL, low-density lipoprotein; VLDL, very low-density lipoprotein.

Based on the patient’s clinical characterizations, which are inconsistent with those of type 1 diabetes, such as the absence of pancreatic antibodies, particularly when measured at diagnosis (9), the persistence of islet function, and the lack of ketoacidosis, as well as the inconsistencies with type 2 diabetes, including the onset of diabetes before the age of 45 years with a normal BMI, the absence of acanthosis nigricans, and normal triglyceride levels, we classify this case as a specific type of diabetes. Moreover, due to the low level of C-peptide and family history, we suspected him to have genetic defects in β cell function such like MODY. With the consent of the patient and his family, we collected peripheral blood samples from the patient, his parents and his son for gene testing.

**Figure 3:**
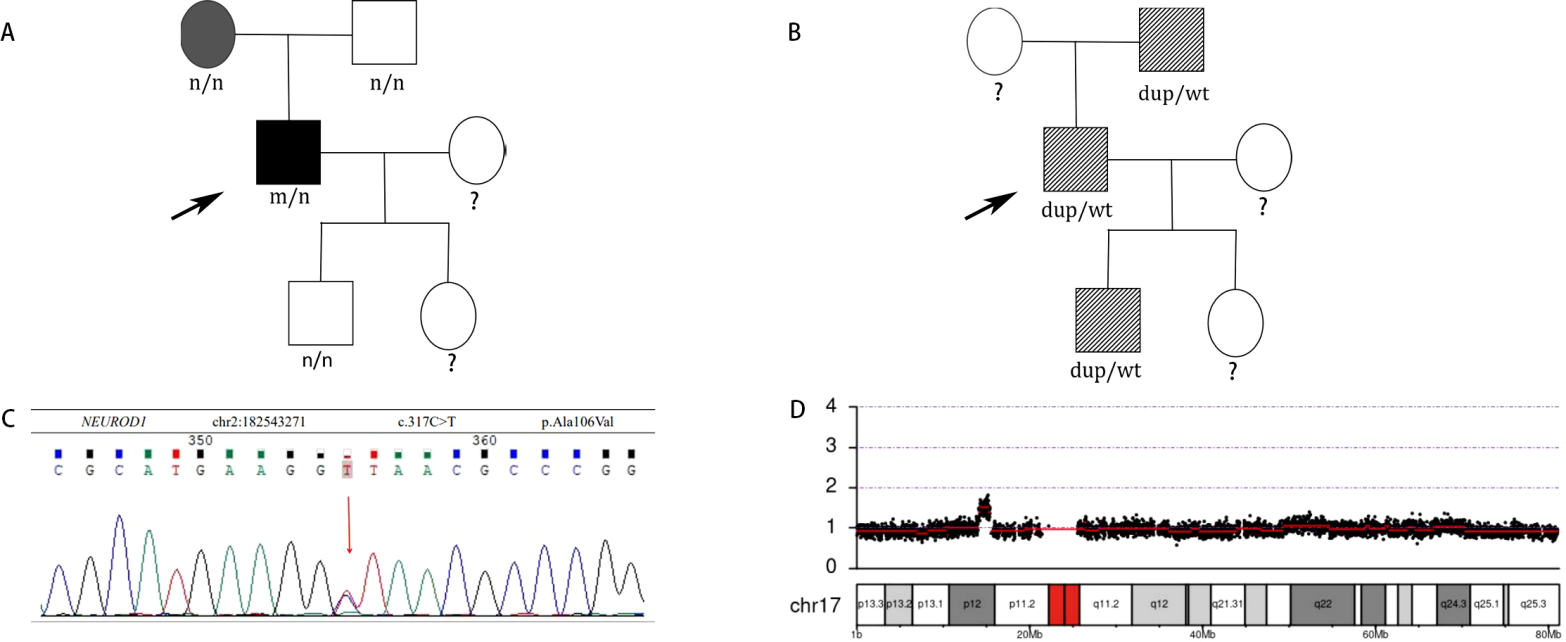
**(A)** Identification of a novel missense *NEUROD1* mutation A106V associated with diabetes in patients. Subjects carrying A106V mutation with diabetes are shown in black. Subjects without carrying A106V mutation with diabetes are shown in gray. Black arrow indicates the probands. n, normal allele; m, mutant allele. **(B)** Identification of *PMP22* duplication associated with CTM1A in patients. Subjects carrying *PMP22* duplication with CMT1A phenotype are shown with slash. Black arrow indicates the probands. wt: wild type, dup: *PMP22* duplication. **(C)** DNA sequences of the *NEUROD1* mutation c.317 C>T found in the patient. **(D)** CNV-seq of the *PMP22* gene duplication on chromosome 17p11.2.

The whole exome sequencing report revealed that a novel heterogeneous missense mutation (NM_002500. 5:c.317C>T, p.Ala106Val) in the exon2 of the *NEUROD1* gene since it has not been reported in the 1000G, gnomAD, or ExAC databases, situated on chromosome chr2:182543271(rs:-) which is PM2 supported, PM3 moderate as shown in **Figures 3A, C**, as well as an approximately 1.38 Mb duplication(number of copies:3) on chromosome 17p11.2 that contains the peripheral myelin protein 22 *(PMP22*) gene which is pathogenic that ClinGen CNV scoring≥ 0.99 causing Charcot-Marie-Tooth disease type 1A (CMT1A) by CNV-seq, which are shown in **Figures 3B, D**. Moreover, the Ala106 residue in the DNA-binding domain of NEUROD1 is evolutionarily conserved in mammals as shown in **Supplementary Figures 1**, **2**, both SIFT and Polyphen algorithms have shown that the Ala106Val mutant is damaging: SIFT score=0 (deleterious), Polyphen score=0.999 (probably damaging) and the PSIPRED analysis indicated that the analyzed substitution appeared to result in helix breakage as shown in **Supplementary Figures 3**, **4**. Treatment protocols: mixed rotamine zinc recombinant human insulin lispro Injection (25R): 18 units (before breakfast), 16 units (before dinner); acarbose tablets: 50mg thrice daily; metformin: 500mg thrice daily; epalrestat: 50mg thrice daily; mecobalamin: 0.5mg thrice daily. After seven days of treatment, the patient’s fasting plasma glucose decreased to 7mmol/L and 2-hour plasma glucose was 6-12 mmol/L.

## 3 Discussion and conclusion

In this study, we described a case of MODY6 associated with a novel mutation in *NEUROD1* within the context of Charcot-Marie-Tooth 1A. The helix-loop-helix (HLH) protein NEUROD1, also known as BETA2, functions as a regulatory switch in the development of the endocrine pancreas (1). Additionally, NEUROD1 binds to the bHLH consensus E-box-binding site within the insulin promoter to activate transcription (3), as well as to the promoters of the sulfonylurea receptor 1 (SUR1) (9), glucokinase (GCK) (10), the glucose-6-phosphatase catalytic subunit-related protein (11), and PAX6 (12), all of these factors contribute to the maintenance of glucose homeostasis. MODY6 represents an extremely rare form of MODY, arising from heterozygous or homozygous mutations in the *NEUROD1* gene (13).

The overall phenotype of MODY6 includes a wide clinical spectrum, ranging from patients displaying typical MODY features to those who exhibit characteristics akin to common type 2 diabetes mellitus (5). The diagnosis of MODY should consider the following atypical features: age under 35 (with age under 25 being more suggestive), negative autoantibodies, the presence of neonatal hypoglycemia, and/or multiple family members with diabetes that does not align with the characteristics of type 1 or type 2 diabetes (14–16). Features atypical for type 1 diabetes mellitus include the following: 1. No presence of pancreatic islet autoantibodies. 2. Demonstrated endogenous insulin production beyond the honeymoon phase. 3. Detectable C-peptide levels during hyperglycemia (C-peptide ≥0.60 ng/mL or 0.2 nmol/L). 4. Low insulin requirements for management (i.e., less than 0.5 U/kg/day). 5. Absence of ketoacidosis when insulin treatment is discontinued. Features atypical of type 2 diabetes mellitus include the following: 1. Onset of diabetes before the age of 45. 2. Absence of significant obesity. 3. Lack of acanthosis nigricans. 4. Normal triglyceride levels and/or normal of elevated high-density lipoprotein cholesterol (HDL-C) (17). Since type 2 diabetes has strong genetic component, shared risk alleles and shared environment that can cause multiple family members to develop type 2 diabetes. In some families, it can be difficult to distinguish from autosomal dominant inheritance. Furthermore, genetic background across different races can significantly influence the pathogenesis of even monogenic forms of diabetes (18). Whole-exome sequencing (WES) has emerged as a powerful tool for discovering novel genes associated with genetic disorders (19). The therapeutic approach for different forms of MODY varies based on their clinical features and underlying molecular causes. In some cases, insulin therapy is required for optimal glucose control, while other patients with MODY can effectively manage their condition with oral hypoglycemic agents (e.g., sulfonylureas) without the need for insulin (20). First-line treatment for MODY6 includes dietary management and oral hypoglycemic agents, such as sulfonylureas, while insulin is considered as second-line therapy (4). The goals of therapy are to enhance quality of life and prevent diabetes-related complications.

The patient’s mother without the mutation in the gene *NEROUD1* has typical characteristics of type 2 diabetes such like: overweight (BMI≥24kg/m2) or obese (BMI≥28kg/m2), onset after age 45, insulin treatment not to be required, abdominal obesity, ketoacidosis seldom occurring spontaneously. We considered the patient’s mother to be a T2DM. However, the patient has clinical characteristics of MODY different from his mother, we considered the novel NEUROD1 heterozygous missense mutation contributed to the clinical features of MODY6. The Ala106Val mutation located in the DNA-binding domain of the NEUROD1 protein impacts the structure of the NEUROD1 to interfere with transcriptional activation. The most probable mechanism is that the side chain of valine is larger than that of alanine and exhibits greater hydrophobicity. Three diabetes pedigrees with different NEUROD1 mutations (Arg111Leu, Glu110Lys and Arg103Pro) at nearby locations have been reported, but their clinical features are different, ranging from MODY to type 2 diabetes, indicating the heterogeneity of the NEUROD1 mutations (1, 21, 22). The whole exome sequencing of family members carrying or not carrying the NEUROD1 mutation, along with functional analyses, could provide insight into how various genetic and/or environmental risk factors collaborate with the NEUROD1 mutation to drive the onset and advancement of diabetes.

Charcot–Marie–Tooth (CMT) disease is the most prevalent inherited neuromuscular disorder (23). The most common genetic form of CMT is Charcot-Marie-Tooth disease type 1A (CMT1A), caused by the duplication of the peripheral myelin protein 22 (PMP22) gene located on chromosome 17 (24, 25), Patients typically exhibit a ‘classical CMT phenotype’ characterized by progressive muscle weakness, atrophy, reduced sensory function, hyporeflexia, and skeletal deformities. The approach to diagnosing CMT is shifting from a purely clinical method in the past to a combined clinical and genetic approach in the present (26). The genetic diagnosis of CMT is performed using targeted next-generation sequencing and whole-exome sequencing techniques. Strategies to downregulate PMP22 gene expression have emerged as a logical extension of treatment research. Some therapies, including ascorbic acid (27), progesterone receptor antagonists (28), small interfering RNA (29) have shown promise in preclinical studies. Unfortunately, the successful translation of these therapies to human trials has been disappointing thus far (30).

PMP22 is expressed in the myelinating Schwann cells of the peripheral nervous system, where it plays an essential role in the formation and maintenance of compact myelin (31). Duplication of the *PMP22* gene leads to a decrease in the amount of functional myelin, causing demyelination and eventually resulting in secondary axonal degeneration and loss during the process of remyelination (32). NEUROD1 is specifically expressed in developing neurons and is crucial for neuronal maturation and neurite outgrowth (33). Interestingly, overexpression of NEUROD1 in adult spinal neurons significantly accelerates axonal regeneration following sciatic nerve injury (34). Based on these findings, we propose that the combination of *NEUROD1* and *PMP22* mutations may exert an additive effect on the nervous system through axonal apoptosis, loss, and regeneration.

In a literature review, we found several reports of patients with CMT1A and diabetes mellitus (DM) (35–39). Reports have documented three families with both CMT1A and type 2 diabetes mellitus (T2DM), suggesting a potential chance association between the two conditions. A retrospective analysis of clinic patients indicated that neuropathy is more severe in diabetic patients with CMT1A compared to those without diabetes (40). A previously reported case of diabetes coexisting with CMT1A, presenting as a recurrent foot ulcer misdiagnosed as a diabetic foot, underscores the importance of differentiating between CMT1A and diabetic peripheral neuropathy (41). The severity of peripheral neuropathy is more significant than other complications such as nephropathy or retinopathy in CMT1A compared to diabetic peripheral neuropathy. However, there are no case reports about CMT patients with the gene mutations of NEUROD1 which are associated with MODY6. Because DM exacerbates motor and sensory impairment in CMT1A, the management of blood glucose is necessary to relieve the peripheral neuropathy.

In conclusion, according to our analysis about clinical features, gene and family history, we considered that the novel mutation of *NEUROD1* is responsible for the diabetes phenotype in the patient. Interestingly and rarely, the patient, his father and his son also are diagnosed with CMT1A. But the patient’s father and son are not diagnosed with DM which are inconsistent with the possibility of a chance association between CMT1A and type 2 DM. However, both of diabetes and CMT1A are likely to have a cumulative negative effective effect on peripheral nerves (38), it is important to differential diagnosis between CMT1A and diabetic peripheral neuropathy and maintain normal blood glucose to relieve peripheral neuropathy. Despite the phenotypic normality observed in his 7-year-old son, it is prudent to focus attention on the peripheral neuromuscular system. Considering the patient’s compromised islet function, our therapeutic approach involved administering insulin to safeguard islet function, metformin to enhance insulin sensitivity, and acarbose to inhibit glucose absorption. The glycaemic control was remarkably improved. There are no FDA-approved treatments for CMT1A. However, further studies will yield insights into the mechanism between the heterozygous mutation of *NEUROD1* gene and diabetes by biology experiments.

## Data Availability

The data sets generated and analyzed during the current study are available from the corresponding author upon reasonable request. The data presented in this study are deposited in the GenBank repository, with accession number PRJNA1221855. The information is accessible at the following link: https://www.ncbi.nlm.nih.gov/sra/PRJNA1221855.
