# Supplementary material for: A case report of a MODY6 patient coexistence with Charcot-Marie-Toothe 1A syndrome
Source: Front Endocrinol (Lausanne). 2025 Feb 14;16:1502783. doi: 10.3389/fendo.2025.1502783 (PMC11867909; doi:10.3389/fendo.2025.1502783)
Supplement: Supplementary file 1 [file DataSheet1.docx]

Supplementary Material


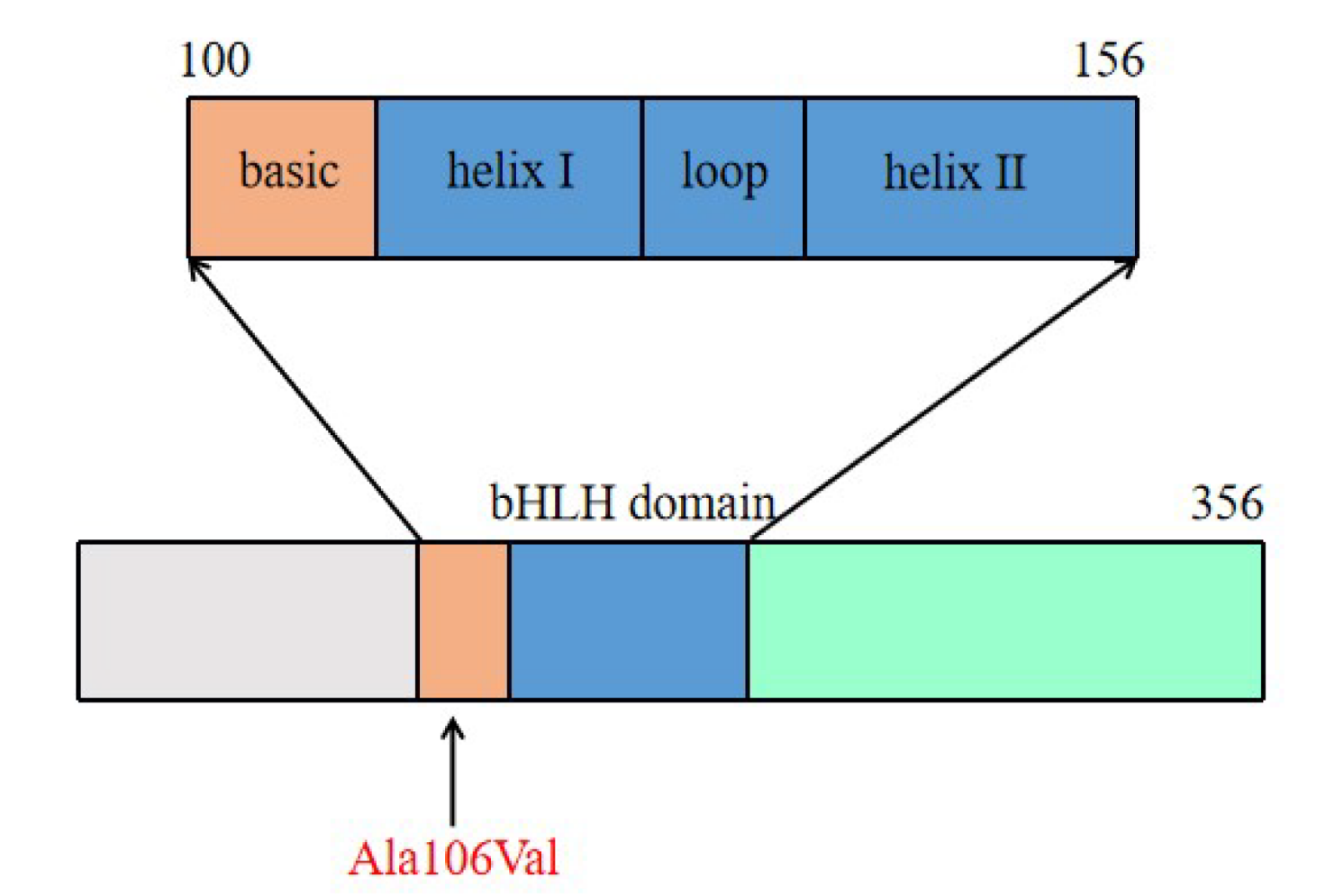


**Supplementary Fig. 1** Schematic organization of NEUROD1 protein. The numbers indicate the amino acids bordering the functional domains. The arrow indicates the identified mutation Ala106Val in NEUROD1 protein.


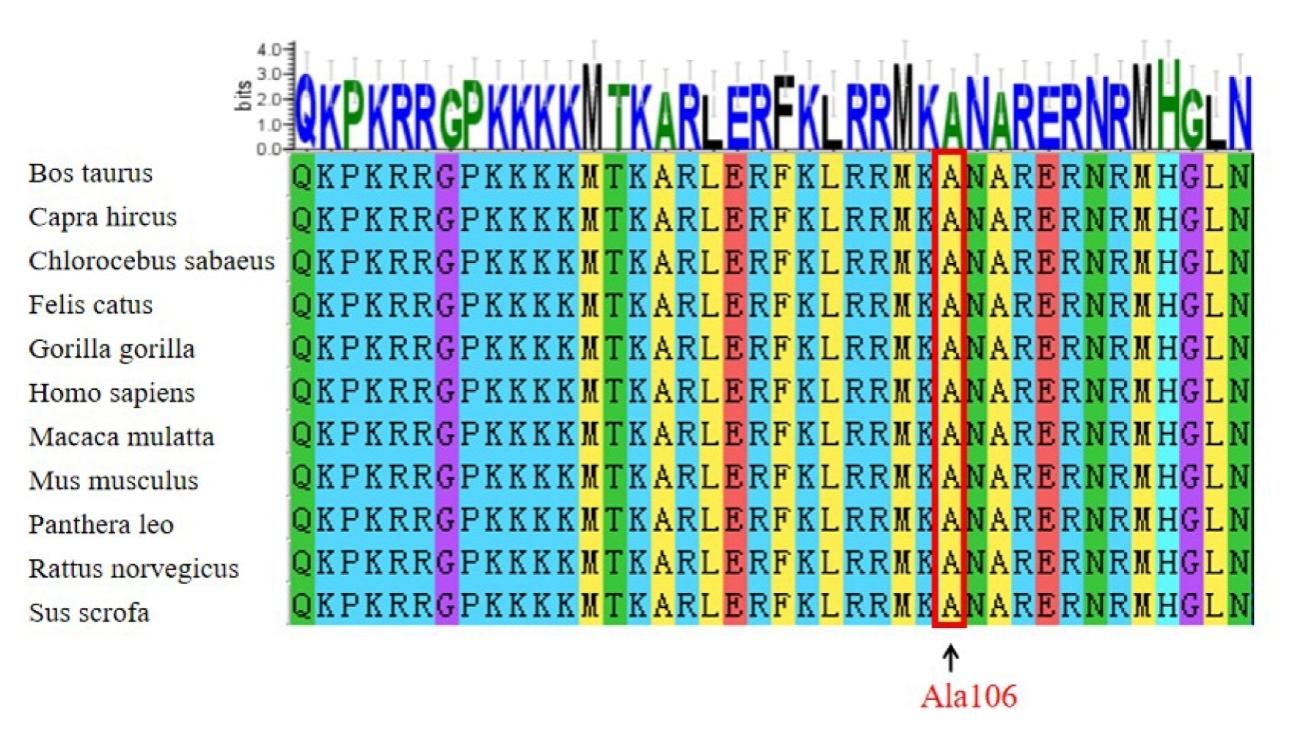


**Supplementary Fig. 2** Alignment of specific regions of NEUROD1 from different mammals using Clustal O. C. The arrow indicates the Ala106 in NEUROD1 protein.


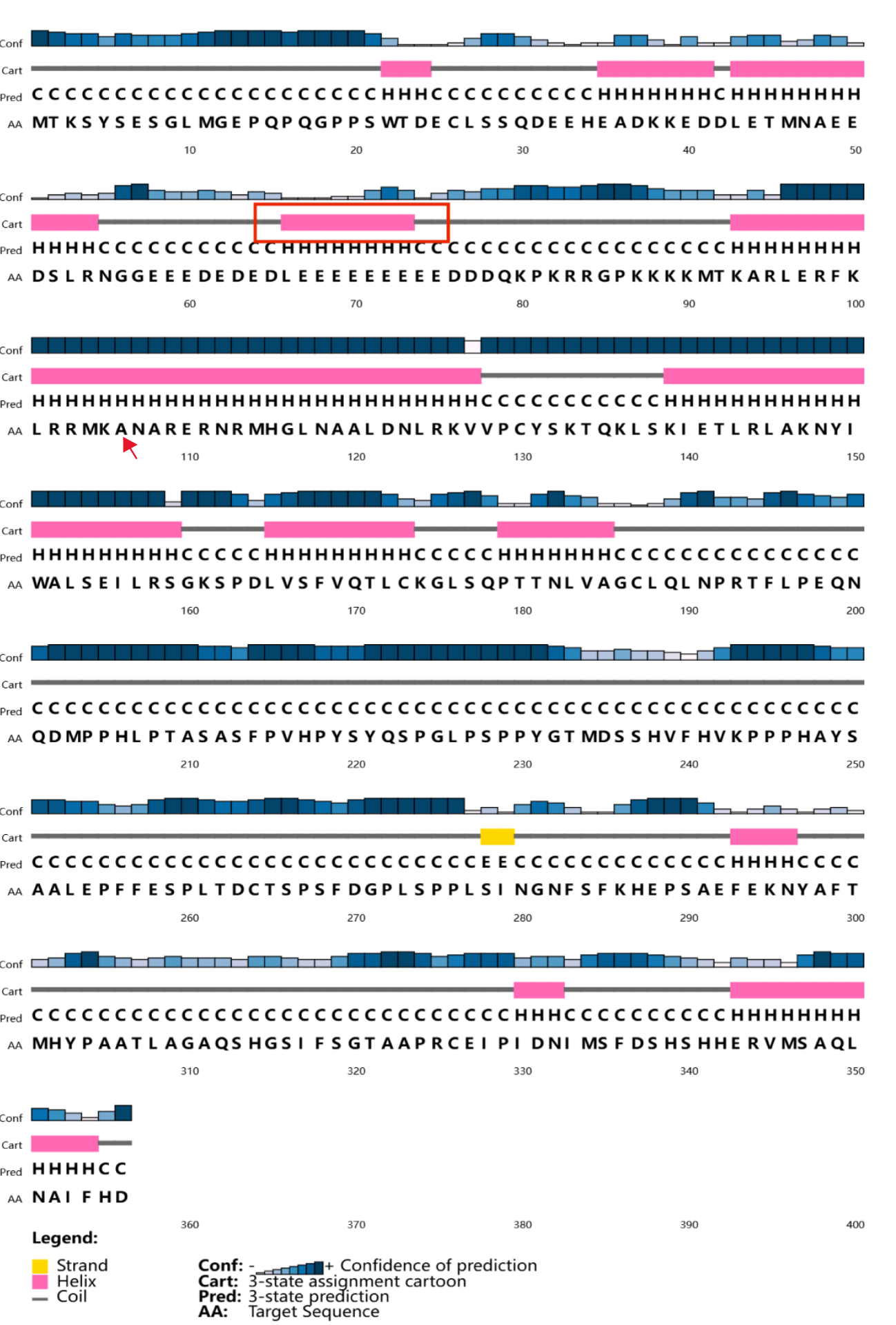


**Supplementary Fig. 3** PSIPRED prediction of the wild-type NEUROD1, the arrow indicates the Ala106Val in NEUROD1, the position of the helix break after mutation is shown in the box.


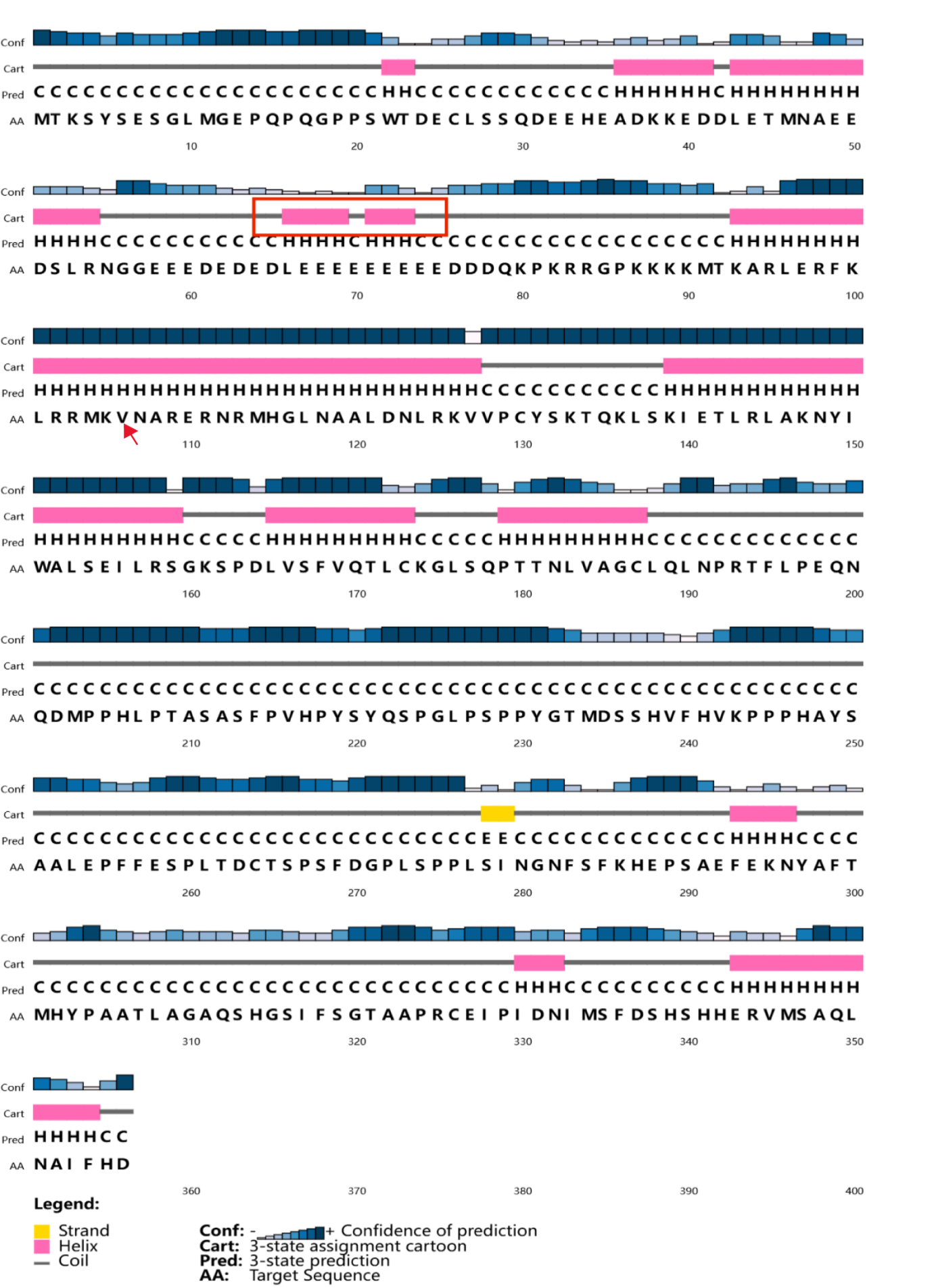


**Supplementary Fig. 4** PSIPRED prediction of the mutation-type NEUROD1, the arrow indicates the Ala106Val in NEUROD1, the position of the helix break after mutation is shown in the box.
